# 

**Published:** 1873-02

**Authors:** 


TWENTY-SEVENTH ANNUAL COMMENCEMENT OF THE BUFFALO MEDICAL COLLEGE.—The commencement exercises of this institution will be held at

St. James Hall, on Tuesday evening, February 25th. The address to the Graduating Class will be delivered by Prof. J. F. Miner. Alumni of the College and all physicians interested in its welfare are invited to be present. The examination before the Curators will take place on Tuesday forenoon at the College building.

### Books and Pamphlets Received.

A Manual of Histology. By Prof. S. Stricker, of Vienna, in co-operation with Th. Meynert, F. Von Recklinhausen, Max Schnltze, W. Waldeyer and others. Translated by Henry Power, of London; Jas. J. Putnam and J. Orne Green, of Boston; Henry C. Eno, Thos. E. Satterthwaite, E. C. Seguin, Lucius D. Bulkley, E. L. Keys and F. E. Delafield, of New York. American translation edited by Albert H. Buck, M. D. New York: Wm. Wood & Co., 1872. Buffalo: H. H. Otis.

Diseases of the Ovaries. Their Diagnosis and Treatment. By T. Spencer Wells. New York: D. Appleton & Co., 1873. Buffalo: Martin Taylor.

Clinical Lectures on Diseases Peculiar to Women. By Lombe Atthill, M. D., Univ. Dub. Second edition, revised and enlarged. Philadelphia: Lindsay & Blakiston, 1873. Buffalo: T. Butler & Son.

Operative Surgery adapted to the Living and Dead Subject. By C. F. Maunder, Surgeon to the London Hospital, etc. Second edition. Philadelphia: Lindsay & Blakiston, 1873. Buffalo: T. Butler & Son.

Fistula, Hæmorrhoids, Painful Ulcer Stricture, Prolapsus, and other Diseases of the Rectum, their Diagnosis and Treatment. By Wm. Allingham, F R. C. S., etc., etc. Second edition, revised and enlarged. Philadelphia: Lindsay & Blakiston, 1873. Buffalo: T. Butler & Son.

A Treatise on the Theory and Practice of Obstetrics. By Wm. H. Byford, A. M., M. D. Second edition, thoroughly revised. New York: Wm. Wood & Co., 1873. Buffalo: H. H. Otis.

A Treatise on Apoplexy, Cerebral Hemorrhage, Cerebral Embolism, Cerebral Gout, Cerebral Rheumatism and Epidemic Cerebro-Spinal Meningitis. By John A. Lidell, A. M., M. D. New York: Wm. Wood & Co., 1873. Buffalo: H. H. Otis.

The Practice of Surgery. By Thomas Bryant, F. R. C. S., Surgeon to Guy Hospital. With two hundred and seven illustrations. Philadelphia: Henry C. Lea, 1873. Buflalo: T. Butler & Son.

Illnstrations of the Influence of the Mind upon the Body in Health and Disease. Designed to Elucidate the Action of the Imagination. By Daniel Hack Tuke, M. D., M. R. C. P. Philadelphia: Henry C. Lea, 1873. Buffalo: T. Butler & Son.

Fœticide, or Criminal Abortion; a lecture introductory to the course on Obstetrics, University of Pennsylvania. By Hugh L. Hodge, M. D. Fourth edition. Philadelphia: Lindsay & Blakiston, 1872. Buffalo: T. Butler & Son.

Half Hour Recreations in Popular Science. Part 6. Unconscious Action of the Brain, and Epidemic Delusions. By Dr W. B. Carpenter, F. R. S. Boston: Estes & Lauriat. Buflalo: H. H. Otis.

Transactions of the Medical Society of the State of West Virginia, June 1872.

THE BUFFALO

Medical and Surgical Journal

PUBLISHED MONTHLY,

Containing Original Articles, Reports of Medical Societies and Hospitals. Editorial Reviews. Correspondence. Army News, etc., etc ; including the usual variety of Medical Periodical Publications. Specimen copies sent on application.

Address Buffalo Medical & Surgical Journal, Buffalo, N. Y.

TERMS OF SUBSCRIPTION, - - $3.00 PER YEAR, IN ADVANCE.

